# RETRACTED: Bucataru et al. Systematic Review and Meta-Analysis of Clinical Efficacy and Safety of Meropenem-Vaborbactam Versus Best-Available Therapy in Patients with Carbapenem-Resistant Enterobacteriaceae Infections. *Int. J. Mol. Sci.* 2024, *25*, 9574

**DOI:** 10.3390/ijms272210123

**Published:** 2026-11-12

**Authors:** Alexandra Bucataru, Adina Turcu-Stiolica, Daniela Calina, Andrei Theodor Balasoiu, Ovidiu Mircea Zlatian, Andrei Osman, Maria Balasoiu, Alice Elena Ghenea

**Affiliations:** 1 Medical Doctoral School, University of Medicine and Pharmacy of Craiova, 200349 Craiova, Romania; alexandra.catana95@gmail.com; 2 Infectious Disease Department, Victor Babes University Hospital Craiova, 200515 Craiova, Romania; 3 Pharmacoeconomics Department, University of Medicine and Pharmacy of Craiova, 200349 Craiova, Romania; 4 Department of Clinical Pharmacy, University of Medicine and Pharmacy of Craiova, 200349 Craiova, Romania; calinadaniela@gmail.com; 5 Department of Ophthalmology, University of Medicine and Pharmacy of Craiova, 200349 Craiova, Romania; andrei_theo@yahoo.com; 6 Department of Bacteriology-Virology-Parasitology, University of Medicine and Pharmacy of Craiova, 200349 Craiova, Romania; ovidiu.zlatian@umfcv.ro (O.M.Z.);; 7 Department of Anatomy and Embryology, Faculty of Dental Medicine, University of Medicine and Pharmacy of Craiova, 200349 Craiova, Romania; 8 Department ENT & Clinical Emergency County Hospital of Craiova, 200642 Craiova, Romania

The journal retracts the article titled, “Systematic Review and Meta-Analysis of Clinical Efficacy and Safety of Meropenem-Vaborbactam versus Best-Available Therapy in Patients with Carbapenem-Resistant Enterobacteriaceae Infections” [1], cited above.

Following its publication, concerns were raised about the meta-analysis which included both the TANGO II randomized trial and a post hoc analysis of a subset of the same trial population, resulting in overlapping datasets and double-counting of patients in this publication [1].

In accordance with the journal’s standard procedures, an investigation was conducted by the Editorial Office and the Editorial Board, that confirmed the presence of overlapping datasets and consequent double-counting of data, resulting in errors in the meta-analysis findings. While the authors collaborated with the investigation and acknowledged the error, the Editorial Board determined that the issues identified exceeded what could be addressed through a standard correction.

As a result, the Editorial Board decided to retract this article in line with MDPI policy on retraction.

This retraction was approved by the Editor-in-Chief of the journal *International Journal of Molecular Sciences*.

The authors have been informed of this retraction and have indicated their agreement with this decision. The authors state that there was an error in the selection and data synthesis.
